# Development and validation of a tool to assess the physical and social environment associated with physical activity among adults in Sri Lanka

**DOI:** 10.1186/1471-2458-14-423

**Published:** 2014-05-03

**Authors:** Shreenika H De Silva Weliange, Dulitha Fernando, Jagath Gunatilake

**Affiliations:** 1 Department of Community Medicine, University of Colombo, Colombo, Sri Lanka; 2 Emeritus Professor of Community Medicine, University of Colombo, Colombo, Sri Lanka; 3 Department of Geology, University of Peradeniya, Peradeniya, Sri Lanka

**Keywords:** Physical and social environment, Physical activity, Development of a tool

## Abstract

**Background:**

Environmental characteristics are known to be associated with patterns of physical activity (PA). Although several validated tools exist, to measure the environment characteristics, these instruments are not necessarily suitable for application in all settings especially in a developing country. This study was carried out to develop and validate an instrument named the “Physical And Social Environment Scale – PASES” to assess the physical and social environmental factors associated with PA. This will enable identification of various physical and social environmental factors affecting PA in Sri Lanka, which will help in the development of more tailored intervention strategies for promoting higher PA levels in Sri Lanka.

**Methods:**

The PASES was developed using a scientific approach of defining the construct, item generation, analysis of content of items and item reduction. Both qualitative and quantitative methods of key informant interviews, in-depth interviews and rating of the items generated by experts were conducted. A cross sectional survey among 180 adults was carried out to assess the factor structure through principal component analysis. Another cross sectional survey among a different group of 180 adults was carried out to assess the construct validity through confirmatory factor analysis. Reliability was assessed with test re-test reliability and internal consistency using Spearman r and Cronbach's alpha respectively.

**Results:**

Thirty six items were selected after the expert ratings and were developed into interviewer administered questions. Exploration of factor structure of the 34 items which were factorable through principal component analysis with Quartimax rotation extracted 8 factors. The 34 item instrument was assessed for construct validity with confirmatory factor analysis which confirmed an 8 factor model (x^2^ = 339.9, GFI = 0.90). The identified factors were infrastructure for walking, aesthetics and facilities for cycling, vehicular traffic safety, access and connectivity, recreational facilities for PA, safety, social cohesion and social acceptance of PA with the two non-factorable factors, residential density and land use mix. The PASES also showed good test re-test reliability and a moderate level of internal consistency.

**Conclusions:**

The PASES is a valid and reliable tool which could be used to assess the physical and social environment associated with PA in Sri Lanka.

## Background

The burden of mortality, morbidity and disability due to non-communicable diseases (NCDs) is high and is increasing in the developing countries [1]. Physical inactivity is identified as the fourth leading risk factor for mortality due to NCDs and contributes to 6% of deaths globally [2]. In 2001, 71% of all deaths in Sri Lanka were due to chronic NCDs and chronic NCD mortality is reported to be 20-30% higher in Sri Lanka than in many developed countries [3]. According to the Annual Health Statistics, coronary heart disease was the leading cause of hospital deaths in Sri Lanka since 1997 [4]. The World Health Survey data collected in 2002–2003 revealed that in Sri Lanka 7.3% of the males and 13.8% of the females were physically inactive [5].

Being physically active is influenced by both the physical and social environment [6,7] and is best explained through a socio-ecological model of health related behaviours. Many studies have recognized that environmental factors have a significant role in promoting PA among adults [8-13] and changing behaviours in an entire community [14]. Literature identifies some common physical (built) environment factors associated with PA. They are land-use patterns, transport systems, urban design, green space, availability of pavements, heavy traffic, street lights, unattended dogs, enjoyable scenery, high levels of crime, and easy access to recreation and retail shops [15,16]. Income, equity, culture and social support are identified in literature as elements in the social environment that influences participation in PA [17,18].

Considering the apparent importance of the environment for PA, there is limited information in the literature on how best to measure various aspects of the environment. Evidence on the associations between the physical environment and PA behaviour is derived mostly from self-reported data on individuals’ perceptions of their environments [19]. Observational methods is another form where individuals using checklists, rates the environment. The introduction of geographic information systems into PA research has revolutionised the measurement of the physical environment, and is still in its early stages [20]. Two major types of PA that have been studied in relation to the environment are the recreational PA and PA through non motorized transportation- walking/cycling. An accepted method of measuring the perceived physical environment is through population based studies and surveillance systems [21]. Individual responses can then be aggregated to identify patterns in environment characteristics. Thereafter, it is possible to determine the association between the design characteristics of the environment and behaviour [22]. There are different tools developed for assessment of environment characteristics that are related to different types of PA.

Abbreviated Neighbourhood Environment Walkability Scale (ANEWS) and the International Physical Activity Questionnaire- environmental (IPAQ-e) are two tools that have been extensively used. ANEWS, a 98 question, self-administered instrument to determine the perception of neighbourhood design features hypothesised to be related to PA [23] was developed in San Diego. It consists of six subscales of land use mix-access, street connectivity, infrastructure for walking/cycling, aesthetics, traffic safety and crime safety [24]. IPAQ-e is a 17 item, 4 factor tool which is considered to be relevant to all countries regardless of the stage of economic development [25], with the factors being the degree of urbanisation, traffic intensity, aesthetics and opportunity and fear of crime [26]. There are several other tools which have been developed in America, Europe and Australia to measure the environment associated with PA. All the above mentioned instruments are known to have an average interviewer administration time of 24–30 minutes [23]. Although tools to assess physical and social environment associated with PA in adults have been developed, validated and utilized extensively in developed countries, South Asia and particularly Sri Lanka lacks a validated tool to assess physical and social environment associated with PA. The purpose of conducting this study was to develop and pilot a tool to assess the physical and social environment associated with PA among adults in the district of Colombo and to assess the construct validity and reliability of it.

## Methods

### Study setting

This study was conducted in the district of Colombo in the western province of Sri Lanka which encompasses the economic capital of Sri Lanka. It extends over an area of 696 square kilometers with a population of 2,390,871 and a population density of 3330 persons per square kilometre [27].

### Arriving at a definition for the physical and social environment associated with PA

Physical and social environment associated with PA was defined initially by considering the definitions given by different authors through a literature search carried out on medical sciences, urban development and design, transport studies and social sciences publications. These definitions were then reviewed with several experts in the fields of community medicine, environment studies, urban planning and architecture, engineering including transport engineering, sociology, health promotion, sports medicine and psychology. The most suitable definition was formulated based on the outcome of the above process.

### Item generation

Item generation was initiated with a literature review which was conducted to identify all aspects of the physical and social environment relevant to PA behaviour among adults in urban and rural communities. Experts who had developed similar questionnaires in the developed countries were contacted. After reviewing several different instruments, all the items used were identified, listed and adopted in a culturally acceptable manner. Thereafter key informant interviews were conducted with the above mentioned experts to generate items. A purposive sampling method was adopted to select the key informants. This was complimented with in-depth interviews with the general public between the ages of 20–59 years living in the Colombo district. Fifteen in-depth interviews were carried out using an interviewer guide. Notes were taken after prior permission. Transcripts of the interviews were made and were coded to identify the main items.

### Analysis of the content of items and item reduction

The items were rated by the experts on a five-point scale (1- least important and 5- most important). An item with a mean score of 3 or more was considered for inclusion in the next round. Items were finalized after two iterations of independent ratings by experts.

### Formulating draft instrument to measure physical and social environment associated with PA and translation

An interviewer administered questionnaire was developed conferring to the measures described by Streiner and Norman [28]. A direct continuous judgment scale with 5 response choices was adopted. The scoring was a simple scoring with scores ranging from 1 to 5. The lowest value 1 indicated the least likelihood of having a conducive environment for PA whereas the highest value 5 indicated the most likelihood of having a conducive environment for PA. The question on residential density and distance to facilities was assessed differently according to the consensus of experts.

The draft instrument developed in English was independently translated to Sinhala by two translators, with a high level of proficiency in English and Sinhala. This was back translated to English and was checked with the original English version and necessary modifications were carried out.

### Finalising the PASES with exploratory factor analysis

In order to assess how the selected 36 items were related to each other, and to see if there was a need for further reduction of items, exploratory factor analysis was carried out using the 34 factorable items. Two items, residential density and land use mix were not included, for the reason that the response categories of these two items were not appropriate to be included in exploratory factor analysis.

Adults aged 20–59 years living in the Colombo district for a period of not less than 6 months were invited to assess their environment using the developed instrument. Institutionalised adults, adults with any physical disability preventing engagement in PA, pregnant females up to a postpartum period of 3 months, adults with severe psychiatric illness and adult visitors to the area were excluded from the study. One hundred and eighty people were interviewed which is more than the recommendation (5 participants per item in the tool) for the sample size in multivariate analysis [29]. A trained interviewer visited the households during weekends to collect data. Data collection was done after information on the purpose of the study was given and written consent was obtained from the selected participant.

Principal Component Analysis (PCA) was applied using Statistical Package for Social Sciences version 17 to explore the factor structure. After assessing the sampling adequacy and factorability, those factors with eigen values of more than one were selected. Scree plots were examined and factors were rotated to optimize the interpretability of the scale. A pre-test was carried out prior to finalizing the instrument -PASES. The PASES is shown as Additional file 1.

### Confirmatory factor analysis

Confirmatory factor analysis (CFA) was carried out to assess the extent to which the underlying eight factor model was replicated in a new data set. Although many factors including size of the model, distribution of variables, amount of missing data, reliability of the variables and strength of the relationship among variables affect the sample size [30], a recommended sample size for CFA is more than 5 times the number of items in the instrument [29]. The instrument was administered to a different group of 180 adults between the ages of 20–59 years living in the Colombo district for a period of not less than 6 months with exclusion criteria similar as above. Data collection too was similar to the data collection procedure for PCA. Data on basic socio demographic and PA were also obtained.

The 34 factorable items were deployed for CFA using LISREL 8.8. after ensuring that the statistical assumptions required for CFA was met. Normality of the data was assessed by inspecting item histograms and calculating the standardized skewness and kurtosis. Multicollinearity was explored through bivariate correlations between the items.

Considering the non-normal distribution of the items of PASES and according to the recommendations offered in LISREL 8.8 [31], Robust Maximum Likelihood (RML) estimation method with the Satorra-Bentler scaled chi-square for fit estimation was used for the CFA. The CFA was performed on the covariance matrix of the items of the PASES. Assessment of the appropriateness of the models was based on several fit indices. The absolute fit indices considered were the χ2 with degrees of freedom (df) and the P value, Goodness of fit index (GFI) and the standardized root mean square residual (SRMR). The Parsimony correlations that were assessed were the root mean square error of approximation (RMSEA). Comparative fit indices that were used were the comparative fit index (CFI) and the Non-normed Fit Index (NNFI) [30]. The judgments about how well the model fit data in this study were made on the basis of RMSEA < 0.05, SRMR < 0.08 CFI > 0.90 and NNFI > 0.90 which have been adopted when assessing model fit in similar situations [32]. The reliability of the instrument was assessed by test re-test method, by administering the same questionnaire to 50 randomly selected participants after a two week interval. Internal consistency which is a measure of “item homogeneity” was assessed by calculating Cronbach’s Alpha for each sub-division score of the PASES.

The Ethics Review Committee of Faculty of Medicine, University of Colombo approved the study protocol (reference number EC-09-084) and data collection was carried out after obtaining informed written consent from each participant.

## Results

### Arriving at a definition for the physical and social environment associated with PA

For the purpose of this study, physical and social environment associated with PA was defined as the “external context consisting of the characteristics of the natural and built environment and the characteristics of the people in the neighbourhood which influences participation in PA”.

### Item generation

The literature review, key informant interviews and in-depth interviews generated 80 items which was reduced to 36 after independent rating by the experts.

### Finalising the PASES with exploratory factor analysis

The sample for the survey to carry out exploratory factor analysis consisted of, 52% males and 48% females. Twenty eight percent of the participants were educated up to G.C.E. O/L or less, while the majority had education above G.C.E. O/L. Majority (84%) of the participants were employed. The sampling adequacy was assessed through inspection of the inter correlation matrix which showed that there were many correlations that were more than 0.3. In the anti-image correlation matrix the coefficients were well above the accepted level of 0.5. Factorability of the data assessed by Bartlett’s test of sphericity, was significant at p < 0.01. The Kaiser- Meyer- Olkin measure was 0.742 which was well above the requirement of 0.6. The items which grouped together were identified as latent factors and were considered relevant only if its eigen value exceeded 1.0. PCA with Quartimax rotation technique gave the best results. Table 1 shows the factor coefficients of individual items after rotation. Eigen values ranged from 7.18 to 1.16. All of the items loaded well to the factors (factor loading >0.4), requiring no further reduction of items. This method initially identified 9 latent factors with one factor retaining only one item. However, as this item also cross loaded with another factor with factor loading of >0.4 and as the cross loading appeared sensible the 8 factor model was selected as the final model after PCA. The factors identified were named as follows:

**Table 1:** Item distribution in PCA (the highest correlation coefficient for the item is in bold)

**Items**	**Infrastructure for walking**	**Aesthetics and facilities for cycling**	**Vehicular traffic safety**	**Access and connectivity**	**Recreational facilities for physical activity**	**Safety**	**Social cohesion**	**Social acceptance of physical activity**
Sidewalks in the main street	**0.788**	−0.076	−0.140	0.095	0.015	−0.240	0.007	0.132
Grass/sand strip in the by roads	**0.573**	0.350	−0.050	−0.084	0.098	−0.316	−0.020	0.099
sidewalks not obstructed	**0.726**	0.183	0.185	0.145	0.031	−0.010	0.131	−0.163
sidewalks free of hazards	**0.666**	0.207	0.101	0.153	0.076	0.315	−0.032	0.007
Special lanes to cycle	0.203	**0.789**	0.083	0.043	0.046	−0.006	−0.088	0.040
Shade in the pathways	−0.001	**0.807**	0.063	0.204	0.066	0.179	0.020	0.088
Trees in the neighbourhood	0.208	**0.508**	0.106	0.383	0.050	0.066	−0.232	0.045
Interesting/pleasant things to look in the neighbourhood	−0.118	**0.589**	0.198	0.189	0.086	0.209	0.129	0.003
Neighbourhood free of dust and fumes	0.206	0.147	**0.560**	0.360	−0.072	−0.092	0.157	−0.112
Low movement of traffic	0.050	0.247	**0.697**	0.313	0.150	0.124	0.052	0.114
Low speed of vehicles	−0.116	0.223	**0.645**	0.328	0.105	0.053	0.323	0.041
Less road traffic accidents	0.092	0.078	**0.560**	0.183	0.051	0.122	0.330	−0.060
Amenities are easily accessible	0.222	0.046	0.215	**0.491**	0.123	−0.006	0.087	0.202
Short distance to main road	−0.203	0.246	−0.314	**0.514**	0.115	−0.115	0.031	0.039
Short distance to transport	−0.015	0.180	0.094	**0.672**	−0.019	−0.228	0.113	0.358
Terrain is good for PA	−0.045	0.013	0.127	**0.575**	0.356	−0.156	0.154	0.092
Presence of pedestrian crossing, signals and overhead bridges	0.118	−0.053	0.116	**0.739**	0.005	0.033	0.322	−0.201
Alternative routes to get from place to place	0.013	0.054	−0.080	**0.638**	−0.013	−0.007	0.228	0.164
Recreational centers for PA	0.040	0.000	−0.003	0.228	**0.573**	0.390	−0.115	0.390
Public spaces for recreation	0.138	0.131	0.024	0.104	**0.474**	0.513	−0.048	0.174
Easy accessibility of recreation places	0.070	0.165	0.061	−0.102	**0.750**	−0.080	0.020	−0.047
Low crime rate	0.201	0.063	0.029	0. 282	−0.109	**0.616**	0.201	−0.074
Well lit roads	−0.117	0.182	0.205	−0.090	−0.005	**0.669**	0.187	−0.217
Neighbourhood free of stray animals	0.122	−0.003	0.110	0.318	−0.134	**0.538**	0.265	−0.040
Good interaction between people in the neighbourhood	0.021	0.138	0.060	−0.016	−0.048	0.385	**0.624**	0.301
Harmony between people in the neighbourhood	0.134	−0.148	0.081	0.145	−0.084	0.047	**0.801**	0.039
Respect each other	−0.082	0.067	−0.018	0.179	0.041	0.150	**0.870**	0.057
Free of social disorder/disputes	−0.062	0.086	0.114	0.135	−0.072	0.064	**0.824**	0.026
Helpful people in the neighbourhood	0.029	−0.087	−0.097	0.192	0.055	−0.185	**0.862**	0.044
Trustworthy people in the neighbourhood	0.081	−0.100	0.054	0.167	0.154	−0.389	**0.696**	0.262
People in the neighbourhood encourage to be active	−0.024	0.059	−0.042	0.018	−0.066	0.020	0.288	**0.687**
People in the neighbourhood are physically active	0.042	−0.001	0.106	0.072	−0.090	−0.060	0.095	**0.854**
Social acceptance for being active for day to day activities	−0.052	0.010	0.063	−0.006	0.005	−0.096	0.068	**0.870**
Social acceptance for Walking, exercising and recreational sport	−0.020	0.103	−0.114	0.006	0.260	0.010	0.064	**0.741**

1 Infrastructure for walking

2 Aesthetics and facilities for cycling

3 Vehicular traffic safety

4 Access and connectivity

5 Recreational facilities for PA

6 Safety

7 Social cohesion

8 Social acceptance of PA

The PASES was validated using CFA for Sri Lanka after the pre-test.

### Confirmatory factor analysis

The response rate of the cross sectional survey was 100%. The socio demographic characteristics of the sample of 180 adults are given in Table 2. The standardised skewness and kurtosis was calculated by dividing the un-standardised skew or kurtosis by its corresponding standard error, which is interpreted as the z test of skew or kurtosis [33]. The ratios greater than 1.96 and have a p value of 0.05, indicate significant skew or kurtosis. The values show that in this sample 22 items had high standard skewness while 10 items had high standard kurtosis. Items in the model should not be highly correlated or perfectly correlated because multicollinearity hinders the interpretability of the results. When the bivariate correlations between the items were examined, although the highest correlation observed between two items was 0.78, 95% of the correlations were less than 0.06 showing that no two items were highly correlated or perfectly correlated. Therefore several models were evaluated using RML method and were assessed for fit indices.

**Table 2:** Frequency distribution of selected socio-demographic characteristics of the participants (n = 180)

**Demographic characteristic**	**Frequency**	**Percentage**
Age category (years)		
20-29	40	22.2
30-39	49	27.2
40-49	43	23.9
50-59	48	26.7
Sex		
Male	78	42.8
Female	102	57.2
Ethnicity		
Sinhalese	177	98.3
Other	03	01.7
Religion		
Buddhism	177	98.3
Other	03	01.7
Monthly family income in Rupees		
10,000 or less	49	27.2
10,001-20,000	90	50.0
20,001-30,000	25	13.9
30,001-40,000	04	02.2
More than 40,000	12	06.7
Presence of a long standing illness of > 6 months		
Yes	40	22.2
No	140	77.8

Initially a 2 factor model was tested where all items in the physical environment were grouped to one and those of the social environment were grouped to another. This model failed to converge and did not show acceptable fit. Thereafter a 6 factor model was tested with ‘infrastructure for walking’ and ‘access and connectivity’ combined as one factor, and vehicular ‘traffic safety’ and ‘safety’ combined as another factor with rest of the factors according to the factors identified by PCA. This model showed acceptable model fit with a chi square value of 364.41(df = 512).

A seven factor model was also tested with item of ‘infrastructure for walking’ and ‘access and connectivity’ combined as one factor. This model showed an acceptable fit with a chi square value of 360 (df = 506). Another 7 factor model with ‘social cohesion’ and ‘social acceptance of PA’ combined together as one factor increased the chi square value to 397 (df = 506). A 8 factor model was tested according to factors derived from PCA. This model showed a better fit with a chi square value of 339.94 (df = 499), p value of 1.00, GFI of 0.90, and RMSEA of 0.001. The summary findings of the model fit statistics of different models are shown in the Table 3. Therefore the 8 factor model was accepted as the best fit model.

**Table 3:** Summary of model fit statistics of the PASES

**Model**	**Absolute fit indices**					**Comparative fit**		**Parsimony correlation**
	**χ2**	**df**	**p**	**GFI**	**SRMR**	**NNFI**	**CFI**	**RMSEA**
6 factor model	364.41	512	1.00	0.89	0.068	1.20	1.00	0.001
7 factor model (a)	360.00	506	1.00	0.89	0.068	1.20	1.00	0.001
7 factor model (b)	397.00	506	0.99	0.88	0.070	1.18	1.00	0.001
8 factor model	339.94	499	1.00	0.90	0.066	1.21	1.00	0.001

χ2 = Chi-square test.

p = (>0.05 desired).

GFI = goodness of fit index (>0.9 desired).

SRMR = standardized root mean square residual (<0.08 desired).

NNFI = Non-normed fit index (>0.9 desired).

CFI = comparative fit index (>0.9 desired).

RMSEA = root mean square error of approximation (<0.05 desired).

The results of the test re-test reliability assessed through Spearman’s r coefficients are given in Table 4. The correlation scores ranged from 0.628-0.916. The lowest correlation 0.628 was in the domains of aesthetics and recreational facility. The internal consistency measured by Chronbach’s Alpha for the physical environment was 0.49, while for the social environment it was 0.82. Both values were significant at p < 0.01 level.

**Table 4:** Test re-test reliability of the mean scores of 8 factors of the PASES

**Factors of PASES**	**Correlation coefficient**
Infrastructure for walking	0.746
Aesthetics and facilities for cycling	0.628
Vehicular traffic safety	0.789
Access and connectivity	0.812
Recreational facilities for PA	0.636
Safety	0.847
Social cohesion	0.916
Acceptance of PA	0.834

## Discussion

An active lifestyle is a complex behavioural process that is influenced by various factors of which environmental factors are well recognized [19,34,35]. This study was designed to develop a valid and reliable tool to assess the physical and social environment associated with PA in the Colombo district, considering the socio-ecological model for PA behaviour.

The procedure adopted to develop PASES was similar to the procedures used for the development of many other study instruments to assess the physical and social environment for PA in other countries [36,37]. The steps included: defining the construct, item generation, analysis of the content of items and item reduction, field testing and validation of the developed instrument [38,39]. Both the quantitative and qualitative research methods provided comprehensive methodologies for exploration of ideas [38], including key informant interviews and in-depth interviews [28]. Item reduction initially was through a simple and non statistical method where a group of experts rated the importance of each item for the appropriateness of the item to the main construct independently. This method facilitated uninfluenced views of each expert as they did not meet each other [40]. According to guidelines of developing new instruments [38], PCA was carried out on a data set that was gathered by administering the translated items to a group of people considered to be similar to the population that the developed instrument was intended to be used [41]. PCA explored the factor structure of the scale and showed that all the items loaded well (factor loadings >0.4) to 8 factors. Hence, no further reduction of data was required through PCA.

CFA on the multi-dimensional construct showed adequate model fit despite emergence of 8 latent factors for the 34 items. Although the PASES was a 8 factor model, the ANEWS had a 6 factor structure after CFA [42] and the IPAQ-e module had 4 factors after PCA [26]. This was expected as some of the items in the three instruments differed. The PASES had two social factors while the ANEWS and IPAQ-e had no social factors.

In the reliability assessment, the Chronbach’s Alpha for physical environment sub-division was 0.487 and for the social environment subdivision was 0.823, indicating a moderate reliability. However, the reliability findings of the present study are comparable with most of reliability tests carried out on environment assessment tools [43,44]. The test re-test reliability in the present study was between 0.6-0.9, confirming the ability of the PASES to generate reproducible results.

The factors identified and validated were comparable with other tools developed in the USA and Europe, with some variation. Infrastructure for walking has been a common factor identified in many instruments [23] and have shown to be associated with the total PA and walking both for leisure and transport [13,45]. Items relating to access and connectivity and the infrastructure for walking were seen to load together as ‘degree of urbanisation’ [26] in the IPAQ-e module but the PASES identified it separately as in the ANEWS [24]. Aesthetics and facilities for cycling were identified as one factor in the PASES as the perception of the beauty of the environment and facilities for cycling were perceived in a similar manner in the Sri Lankan setting. However aesthetics were a separate factor in both the ANEWS and IPAQ-e tools. Vehicular traffic safety, and safety which are important factors in the environment, were identified in the newly developed and validated tool, with additional factor of ‘recreational facilities for PA’. Two social factors, namely social cohesion and social acceptance of PA were identified as important and were incorporated to the PASES. Although not factor analysed ‘residential density’ and ‘land use mix diversity’ were components of the newly developed tool PASES. These two components measure proximity, indicating how close different travel destinations are to one another in space. Density indicates the concentration of people, dwelling units or households [45] and mixture of use of land refers to the spatial placement of different types of land uses (industrial, residential, commercial). The factors identified in the PASES were in some ways similar to other environmental assessment tools with some variation which might reflect the Asian setting.

However, it should be noted that findings of factor analysis assessments are often sample specific [46] and generalizability of these findings to other populations would depend on their similarity to this study population. The specific “aspects” that could be studied may vary from within and between countries. This study instrument could be used to assess the physical and environmental factors associated with PA in South Asia and other parts of Sri Lanka after testing for reliability and validity in that particular setting.

## Conclusion

A valid and reliable tool to assess the physical and social environment associated with PA was developed in Sri Lanka. This work contributes to a set of tools which can be used by researchers to identify the current perception of the environment for participation in PA by the community and to assess any change in the perception with interventions and with time.

## Abbreviations

ANEWS: Abbreviated neighbourhood environment walkability scale; CFA: Confirmatory factor analysis; CFI: Comparative fit index; GFI: Goodness of fit index; IPAQ-e: International physical activity questionnaire –environment; NCD: Non communicable disease; NNFI: Non-normed fit index; PA: Physical Activity; PASES: Physical and social environment scale; RMSEA: Root mean square error of approximation; SRMR: Standardized root mean square residual.

## Competing interests

The authors declare that they have no competing interests.

## Authors’ contributions

SDW – made contribution to the design, planning the study, literature search, acquisition of data, analysis and interpretation of the results, drafting the manuscript and revising it critically and has given final approval of the version to be published. DF – contributed to the planning of the study, assisted in planning data analysis, drawing conclusions and in revising it critically for important intellectual content the preparation of the research paper and has given final approval of the version to be published. JG – contributed to the planning of the study, planning data analysis, drawing conclusions and in revising it critically for important intellectual content the preparation of the research paper and has given final approval of the version to be published.

## Authors’ information

SDW is a lecturer in the Department of Community Medicine, University of Colombo. DF is the Emeritus Professor of Community Medicine, University of Colombo and JF is a senior lecturer in the Department of Geology, University of Peradeniya and is the coordinator, Msc programme in GIS and Remote Sensing at the University of Peradeniya Sri Lanka.

## Pre-publication history

The pre-publication history for this paper can be accessed here:

http://www.biomedcentral.com/1471-2458/14/423/prepub

## Supplementary Material

Additional file 1 Physical and Social Environment Scale-PASES. Click here for file

## References

[B1] World Health Organisation Global strategy on diet, physical activity and health 2004 [http://www.who.int/dietphysicalactivity/strategy/eb11344/strategy_english_web.pdf]

[B2] World Health Organisation Global status report on non-communicable diseases 2010 [http://www.who.int/nmh/publications/ncd_report_full_en.pdf]

[B3] World Bank Sri Lanka- Addressing needs of an aging population [http://www-wds.worldbank.org/external/default/WDSContentServer/WDSP/IB/2010/10/29/000334955_20101029044915/Rendered/PDF/575630WP0Box351DS0SriLanka01PUBLIC1.pdf]

[B4] Ministry of Healthcare and Nutrition Sri Lanka Annual Health Statistics 2003 Colombo: Ministry of Healthcare and Nutrition

[B5] Guthold R Ono T Strong KL Chatterji S Morabia A Worldwide variability in physical inactivity: a 51- country survey Am J Prev Med 2008 34 6 486 494 10.1016/j.amepre.2008.02.013 18471584

[B6] Sallis JF Cervero RB Ascher W Henderson KA Kraft MK Kerr J An ecological approach to creating active living communities Annu Rev Publ Health 2006 27 297 322 10.1146/annurev.publhealth.27.021405.102100 16533119

[B7] Stokols D Translating social ecological theory into guidelines for community health promotion Am J Health Promot 1996 10 282 298 10.4278/0890-1171-10.4.282 10159709

[B8] Troped PJ Saunders RP Pate RR Reininger B Ureda JR Thompson SJ Associations between self-reported and objective physical environmental factors and use of a community rail-trail Prev Med 2001 32 2 191 200 10.1006/pmed.2000.0788 11162346

[B9] Handy SL Critical assessment of the literature on the relationships among transportation, land use, and physical activity TRB special report 282 2005 Washington, DC: Transportation Research Board and Institute of Medicine Committee on Physical Activity, Health, Transportation, and Land Use

[B10] Owen N Humpel N Leslie E Bauman A Sallis J Understanding environmental influences on walking: review and research agenda Am J Prev Med 2004 27 67 76 10.1016/j.amepre.2004.03.006 15212778

[B11] Owen N Leslie E Salmon J Fotheringham M Environmental determinants of physical activity and sedentary behavior Exerc Sports Sci Rev 2000 28 153 158 11064848

[B12] Saelens B Sallis J Frank L Environmental correlates of walking and cycling: findings from the transportation, urban design and planning literatures Ann Behav Med 2003 25 80 91 10.1207/S15324796ABM2502_03 12704009

[B13] Duncan MJ Spence JC Mummery WK Perceived environment and physical activity: a meta-analysis of selected environmental characteristics Int J Behav Nutr Phys Activ 2005 2 11 10.1186/1479-5868-2-11 PMC1236952 16138933

[B14] Sallis JF Owen N Physical activity and behavioral medicine 1999 Thousand Oaks, CA: Sage Publications Inc

[B15] Frank LD Engelke P Multiple impacts of the built environment on public health: walkable places and the exposure to air pollution Int Reg Sci Rev 2005 28 2 193 216 10.1177/0160017604273853

[B16] Addy CL Wilson DK Kirtland KA Ainsworth BE Sharpe P Kimsey D Associations of perceived social and physical environmental supports with physical activity and walking behaviour Am J Publ Health 2004 94 3 440 443 10.2105/AJPH.94.3.440 PMC1448272 14998810

[B17] Ishii K Shibata A Oka K Environmental, psychological, and social influences on physical activity among Japanese adults: structural equation modeling analysis Int J Behav Nutr Phys Activ 2010 7 61 10.1186/1479-5868-7-61 PMC2927494 20684794

[B18] Stahl T Rütten A Nutbeam D Bauman A Kannas L Abel T Lüschen G Rodríguez Diaz JA Vinck J van der Zee J The importance of the social environment for physically active lifestyle-results from an international study Soc Sci Med 2001 52 1 1 10 10.1016/S0277-9536(00)00116-7 11144909

[B19] Humpel N Owen N Leslie E Environmental factors associated with adults’ participation in physical activity: a review Am J Prev Med 2002 22 3 188 199 10.1016/S0749-3797(01)00426-3 11897464

[B20] SallisJ F Measuring physical activity environments-a brief history Am J Prev Med 2009 36 4S 86 92 10.1016/j.amepre.2009.01.002 PMC2921817 19285214

[B21] Baker EA Brennan LK Brownson R Houseman RA Measuring the determinants of physical activity in the community: current and future directions Res Q Exerc Sport 2000 71 2S 146 158 10.1080/02701367.2000.11082798 25680025

[B22] Brownson RC Baker EA Housemann RA Brennan LK Bacak SJ Environmental and policy determinants of physical activity in the United States Am J Publ Health 2001 91 12 1995 2003 10.2105/AJPH.91.12.1995 PMC1446921 11726382

[B23] Brownson RC Chang JJ Eyler AA Ainsworth BE Kirtland KA Saelens BE Sallis JF Measuring the environment for friendliness towards physical activity: a comparison of the reliability of 3 questionnaires Am J Publ Health 2004 94 3 473 483 10.2105/AJPH.94.3.473 PMC1448279 14998817

[B24] Cerin E Saelens BE Sallis JF Frank LD Neighbourhood environment walkability scale: validity and development of a short form Med Sci Sports Exerc 2006 38 9 1682 1691 10.1249/01.mss.0000227639.83607.4d 16960531

[B25] Adegoke BOA Oyeyemi AY Fatudimu BM Test-retest reliability of IPAQ environmental-module in an African population International Journal of Behavioral Nutrition and Physical Activity 2008 5 38 44 10.1186/1479-5868-5-38 18680599 PMC2531132

[B26] Bergman P Grjibovski AM Hagströmer M Sallis JF Sjöström M The association between health enhancing physical activity and neighbourhood environment among Swedish adults – a population-based cross-sectional study Int J Behav Nutr Phys Activ 2009 6 8 10.1186/1479-5868-6-8 PMC2647520 19203354

[B27] Department of Census and Statistics Sri Lanka Census of population and housing-2001 Sri Lanka: Colombo district report 2004 Colombo: Department of Census and statistics

[B28] Streiner DL Norman GR Health measurement scales: a practical guide to their development and use 1995 3 Oxford: Oxford University Press

[B29] Tabachnick BG Fidell LS Using multivariate statistics 1989 2 London: Harper & Row

[B30] Brown TA Confirmatory factor analysis for applied research 2006 New York: Guilford

[B31] JöreskogK G Sörbom D LISREL 8: User’s reference guide 1996 Chicago, IL: Scientific Software International

[B32] Chang M Brown R Nitzke S Scale development: factors affecting diet, exercise, and stress management (FADESM) BMC Public Health 2008 8 76 10.1186/1471-2458-8-76 18302762 PMC2266923

[B33] Kline RB Principles and Practice of Structural Equation Modeling 2005 2 New York: The Guilford Press

[B34] Popkin BM Duffey K Gorden-Larsen P Environmental influences on food choice, physical activity and energy balance Physiol Behav 2005 86 5 603 613 10.1016/j.physbeh.2005.08.051 16246381

[B35] De Bourdeaudhuij I Sallis JF Saelens BE Environmental correlates of physical activity in a sample of Belgian adults American Journal of Health Promotion 2003 18 1 83 92 10.4278/0890-1171-18.1.83 13677966

[B36] McCormack GR Masse LC Bulsara M Pikora TJ Giles-Corti B Constructing indices representing supportiveness of the physical environment for walking using the Rasch measurement model Int J Behav Nutr Phys Activ 2006 3 44 10.1186/1479-5868-3-44 PMC1764897 17173695

[B37] Spittaels H Foster C Oppert JM Rutter H Oja P Sjöström M De Bourdeaudhui I Assessment of environmental correlates of physical activity: development of a European questionnaire Int J Behav Nutr Phys Activ 2009 6 39 10.1186/1479-5868-6-39 PMC2713198 19580645

[B38] Redding CA Maddock JE Rossi JS The sequential approach to measurement of health behavior constructs: issues in selecting and developing measures CA J Health Promot 2006 4 1 83 101

[B39] Yousefian A Hennessy E Umstattd RM Economos CD Hallam JS Hyatt RR Hartley D Development of the rural active living assessment tools: measuring rural environments Prev Med 2010 50 S86 S92 19818362 10.1016/j.ypmed.2009.08.018

[B40] Snyder-Halpern R Indicators of organizational readiness for clinical information technology/ systems innovation: a Delphi study Int J Med Informat 2001 63 179 204 10.1016/S1386-5056(01)00179-4 11502432

[B41] Ogilvie D Mitchell R Mutrie N Petticrew M Platt S Perceived characteristics of the environment associated with active travel: development and testing of a new scale Int J Behav Nutr Phys Activ 2008 5 32 10.1186/1479-5868-5-32 PMC2416440 18513430

[B42] LeslieE B Saelens BE Frank LD Owen N Bauman A Coffee N Hugo G Residents’ perception of walkability attributes in objectively different neighbourhoods: a pilot study Health Place 2005 11 227 236 10.1016/j.healthplace.2004.05.005 15774329

[B43] Saelens BE Sallis JF Black JB Chen D Neighbourhood- based differences in physical activity: an environment scale evaluation Am J Publ Health 2003 93 9 1552 1558 10.2105/AJPH.93.9.1552 PMC1448009 12948979

[B44] Rhodes RE Courneya KS Blanchard CM Plotnikoff RC Prediction of leisure-time walking: an integration of social cognitive, perceived environmental and personality factors Int J Behav Nutr Phys Activ 2007 4 51 10.1186/1479-5868-4-51 PMC2174941 17974022

[B45] Handy SL Boarnet MG Ewing R Killingsworth RE How the built environment affects physical activity: views from urban planning Am J Prev Med 2002 23 2S 64 73 12133739 10.1016/s0749-3797(02)00475-0

[B46] MacCallum RC Widaman KF Zhang S Hong S Sample size in factor analysis Psychol Meth 1999 4 1 84 99

