# Supplementary material for: Development and validation of a tool to assess the physical and social environment associated with physical activity among adults in Sri Lanka
Source: BMC Public Health. 2014 May 3;14:423. doi: 10.1186/1471-2458-14-423 (PMC4021224; doi:10.1186/1471-2458-14-423)
Supplement: Additional file 1 — Physical and Social Environment Scale-PASES. [file 1471-2458-14-423-S1.pdf]

Physical And Social Environment Scale – PASES

We would like to find out information about the way that you perceive or think about your neighbourhood in relation to physical activity. Please express **what you think or feel** about the different facilities in and around your neighbourhood.

Neighbourhood is defined as the area ALL around your home that you could walk to in 10 minutes.

Within walking distance ,means it is within a 10 minute walk from your home.

Please mark with a tick '✓' the relevant box that best applies to you and your neighborhood.

Part 1-Physical Environment

Now we want information about the physical environment.

1.1 Residential Density

What is the commonest type of residences in your neighborhood**? (single response)**

Detached single residences	1
Annexed row houses or 2-3 storied flats or apartments	2
Mix of 1 and 2 above	3
Apartments or flats 4-12 stories	4
Apartments or flats more than 12 stories	5
Do not know	88

1.2-1.7 To what extent do you agree with the following statements about your neighbourhood in relation to physical activity?

**1.2 Regarding streets in the neighbourhood**		1	2	3	4	5
		**Strongly disagree**	**Disagree to a certain extent**	**Neither agree nor disagree**	**Agree to a certain extent**	**Agree strongly**
**Q1.**	There are sidewalks on most of the main streets in my neighbourhood	1	2	3	4	5
**Q2.**	The sidewalks available along the main streets in my neighbourhood are obstructed (parked vehicles, pavement hawkers, garbage etc).	1	2	3	4	5
**Q3.**	The sidewalks available in my neighbourhood are hazardous for walking due to pot holes, open drains and cracked/ uneven surfaces etc.	1	2	3	4	5
**Q4.**	There is a separate grass/sand strip for walking in the by-roads in my neighbourhood	1	2	3	4	5
**Q5.**	There are special lanes, separate paths, trails or special areas allocated to bicycling in my neighbourhood	1	2	3	4	5

**1.3 Regarding how pleasant your neighbourhood surroundings are?**		1	2	3	4	5
		**Strongly disagree**	**Disagree to a certain extent**	**Neither agree nor disagree**	**Agree to a certain extent**	**Agree strongly**
**Q6.**	There is shade in my neighbourhood	1	2	3	4	5
**Q7.**	There are **NO** big trees in my neighbourhood.	1	2	3	4	5
**Q8.**	There are many useful, attractive things to look at while walking/cycling in my neighbourhood.(natural sights, beautiful buildings)	1	2	3	4	5

**1.4 Regarding access to places in the neighbourhood**		**1**	**2**	**3**	**4**	**5**
		**Strongly disagree**	**Disagree to a certain extent**	**Neither agree nor disagree**	**Agree to a certain extent**	**Agree strongly**
**Q9.**	There are many alternative routes for getting from place to place in my neighbourhood. (I don't have to go the same way every time.)	1	2	3	4	5
**Q10.**	Grocery stores, super markets, shops and many places that need to be visited day to day are #**within walking distance of my home.**	1	2	3	4	5
**Q11.**	Can walk to the main road within 10 minutes from my home.	1	2	3	4	5
**Q12.**	Can walk to a transit stop like bus or train within 10 minutes from my home.	1	2	3	4	5
**Q13.**	Since my neighborhood is hilly, it is difficult to walk in the streets.	1	2	3	4	5

**1.5. Regarding vehicular traffic in the neighbourhood**		**1**	**2**	**3**	**4**	**5**
		**Strongly disagree**	**Disagree to a certain extent**	**Neither agree nor disagree**	**Agree to a certain extent**	**Agree strongly**
**Q14.**	Walking is difficult since my neighbourhood environment is polluted with vehicle fumes and dust	1	2	3	4	5
**Q15.**	In my neighbourhood there is **NOT** much vehicular traffic to the extent of making it difficult to walk on the streets	1	2	3	4	5

**Q16.**	Most drivers **do not** exceed speed limit in my neighbourhood	1	2	3	4	5

**1.6 Regarding facilities for engaging in physical activity in the neighbourhood**		**1**	**2**	**3**	**4**	**5**
		**Strongly disagree**	**Disagree to a certain extent**	**Neither agree nor disagree**	**Agree to a certain extent**	**Agree strongly**
**Q17.**	My neighbourhood has several **free** or **very low cost**, centers for sports and exercise	1	2	3	4	5
**Q18.**	There are many public places for sports and recreation in my neighbourhood(parks, beach etc)	1	2	3	4	5
**Q19.**	There is easy access to the public places for sports and recreation in my neighbourhood (parks, beach etc)	1	2	3	4	5

**1.7 Regarding neighbourhood safety**		**1**	**2**	**3**	**4**	**5**
		**Strongly disagree**	**Disagree to a certain extent**	**Neither agree nor disagree**	**Agree to a certain extent**	**Agree strongly**
**Q20.**	Many road traffic accidents occur in my neighbourhood	1	2	3	4	5
**Q21.**	The crime rate and insecure state in my neighbourhood make it **unsafe** to go on walks	1	2	3	4	5
**Q22.**	My neighbourhood streets are **NOT** well lit by street lamps at night.	1	2	3	4	5
**Q23.**	Walking is **DANGEROUS** due to the presence of stray dogs, snakes and other troublesome animals in my neighbourhood	1	2	3	4	5
**Q24.**	There are **NO** pedestrian crossings, signals and overhead bridges to help walkers cross busy streets in my neighbourhood	1	2	3	4	5

1.8 Land use diversity

Now we are asking about your ability to go to various places in your neighbourhood.

About how long would it take to go from your home to the nearest facilities listed below if you walked to them? (**Single response)**

Facility		1	2	3	4	5
		**1-5 minutes**	**6-10 minutes**	**11-20 minutes**	**20-30 minutes**	**>30 minutes**
1.	Convenience/grocery store	1	2	3	4	5
2.	Supermarket	1	2	3	4	5
3.	Hardware store	1	2	3	4	5
4.	Fruit/vegetable market	1	2	3	4	5
5.	Clothing store	1	2	3	4	5
6.	Post office	1	2	3	4	5
7.	School	1	2	3	4	5
8.	Bakery, restaurant	1	2	3	4	5
9.	Bank	1	2	3	4	5
10.	Pharmacy/drug store	1	2	3	4	5
11.	Salon/barber shop	1	2	3	4	5
12.	Bus or train stop	1	2	3	4	5
13.	Public places like Park, Beach	1	2	3	4	5
14.	Gymnasium/sports/recreation center	1	2	3	4	5

Part 2 - Social Environment

Now we are asking about the social environment.

2.1- 2.2 To what extent do you agree with the following statements?

**2.1 Regarding social cohesion**		1	2	3	4	5
		**Strongly disagree**	**Disagree to a certain extent**	**Neither agree nor disagree**	**Agree to a certain extent**	**Agree strongly**
**Q25.**	There is **NO** harmony between people in my neighbourhood	1	2	3	4	5
**Q26.**	People in my neighbourhood respect each other	1	2	3	4	5
**Q27.**	There are social disputes among the people in my neighbourhood	1	2	3	4	5

**Q28.**	People in my neighbourhood are willing to help each other in time of need	1	2	3	4	5
**Q29.**	People in my neighbourhood are **NOT** trustworthy	1	2	3	4	5
**Q30.**	There are **NO** satisfactory social interactions and strong social networks among the people in my neighbourhood.	1	2	3	4	5

**2.2 Regarding social acceptance of physical activity**		**1**	**2**	**3**	**4**	**5**
		**Strongly disagree**	**Disagree to a certain extent**	**Neither agree nor disagree**	**Agree to a certain extent**	**Agree strongly**
**Q31.**	People in my neighbourhood are very active	1	2	3	4	5
**Q32.**	The people in my neighbourhood encourage me for active living	1	2	3	4	5
**Q33.**	There is **NO** social acceptance among the people in my neighbourhood for walking, doing moderate and vigorous physical activities for day to day work	1	2	3	4	5
**Q34.**	There is social acceptance among the people in my neighbourhood for walking, /jogging, running, exercising for recreation and sports	1	2	3	4	5

Thank you.
